# Association between self-esteem and efficacy and mental health in people with disabilities

**DOI:** 10.1371/journal.pone.0257943

**Published:** 2021-10-06

**Authors:** Jong Youn Moon, Jae-Hyun Kim

**Affiliations:** 1 Department of Preventive Medicine, Gachon University College of Medicine, Incheon, Republic of Korea; 2 Center for Public Health, Gachon University Gil Medical Center, Incheon, Republic of Korea; 3 Department of Health Administration, College of Health Science, Dankook University, Cheonan, Republic of Korea; 4 Institute for Digital Life Convergence, Dankook University, Cheonan, Republic of Korea; University of Perugia: Universita degli Studi di Perugia, ITALY

## Abstract

This study aimed to investigate the association among self-evaluations—such as self-esteem and self-efficacy—self report of depression, and perceived stress among Korean individuals with disabilities. Data from the second wave of the Panel Survey of Employment for the Disabled (collected from 2016–2018) were used. In 2016 and the follow-up in 2018, 4,033 participants were included. We estimated the annual change in both independent variables and the probability of self-report of depression and stress. Generalized estimating equation model and chi-square test were used. Compared with those whose self-esteem and self-efficacy scores were ≥30, those with scores ≤19 were, respectively, 5.825 (95% Confidence Interval [*CI*]: 4.235–8.011; *p* < .0001) and 1.494 times (95% *CI*: 1.233–1.810; *p* < .0001) more likely to have self-report of depression. The perceived stress of those with self-esteem scores ≤19 or ranging from 20–24 were, respectively, 2.036 (95% *CI*: 1.510–2.747; *p* < .0001) and 1.451 times higher (95% *CI*: 1.269–1.659; *p* < .0001) than those with self-esteem scores ≥30. There exists an inverse correlation between self-evaluations, such as self-efficacy and self-esteem, and mental health in people with disabilities. The results of this study can be used as a basis for developing interventional strategies and training and intervention programs for people with disabilities. Future research is needed to investigate potential mediating factors among Korean individuals.

## Introduction

Given an aging population, the number of people with disabilities has increased globally in recent decades [1]. In South Korea, registered individuals with disabilities accounted for 2.4% of the total population in 2001; this gradually increased to 4.9% in 2016. The reason for this increase is thought to be a change in the demographic structure. Korea has one of the lowest fertility rates in the world and is rapidly aging. Since 2013, more than 40% of registered individuals with disabilities in Korea are 65 years old or older [2]. Accordingly, people with physical or mental disabilities face many health issues, such as psychosocial stress [3] and symptoms of depression [4].

Among individuals with disabilities, self-evaluation related to the day-to-day management of their condition is of interest. Self-efficacy and self-esteem, which are psychological constructs influencing the management of stress and depression [5, 6], can be conceived as components of core self-evaluations. These are fundamental assumptions an individual makes about themselves and their agency within their environment [7]. More specifically, self-efficacy is the belief of an individual that they will be able to perform specific behaviors in particular situations that may contain novel, unpredictable, and stressful elements [8]. Self-esteem consists of the positive or negative attitudes of an individual toward themselves; meanwhile, specific self-esteem refers to attitudes toward the self in distinct contexts, such as academia [9].

Self-evaluation produces diverse effects on human functioning through major psychological processes, including cognitive, motivational, selection, and affective processes that are considered reciprocal interactions between environmental, behavioral, and personal factors [10]. By influencing affective processes, self-evaluation plays an essential role in the physical and mental health of an individual. High self-evaluation is considered a beneficial psychological resource, leading to higher life satisfaction, greater psychological wellbeing, improved mental health, and less anxiety and depression in people with disabilities [10, 11]. Additionally, high self-evaluation helps create feelings of serenity when approaching difficult tasks and activities [12, 13].

Currently, a substantial amount of research [14, 15]—including meta-analytic evidence—suggests that core self-evaluations, such as self-esteem and self-efficacy, are associated with mental health outcomes and life satisfaction. The relationship between self-efficacy and depression is explored among different samples (e.g., patients with various disorders [16], individuals suffering from some type of injury or pain [17], or elderly individuals [18]).

There is still a lack of understanding of the specific associations between self-evaluations, such as self-efficacy and self-esteem, and mental health among people with disabilities in South Korea. Given this, the present study aimed to use an evidence-based approach to understand the association between self-evaluations, such as self-efficacy and self-esteem, and mental health among the people with disability.

## Methods

### Data source

This study used data from the second wave—from 2016–2018—of the Panel Survey of Employment for the Disabled (PSED) conducted by the Korea Employment Agency for the Disabled. For the construction of the second wave panel, the target population was those 15–64 years old; this age group was chosen since they are representative of the core productive population as registered persons with disabilities under the Disability Welfare Act. The PSED repeatedly measured panel data from households, including those with individuals with disabilities; it provides useful data for understanding the economic activities of people with disabilities with regard to their employment. People with disabilities were interviewed individually in the PSED. Given that the structure and content of these data are more complex than cross-sectional data, this survey used a computer-assisted personal interviewing method to perform “logic checks” to identify inconsistent or contradictory responses by Korea Employment Agency for the Disabled. The PSED only allowed the head of the household or the legal guardian to reply if an intellectual disability or mental disorder limited direct communication. As for the number of participants, 4,577 participants joined the baseline survey in 2016. The second survey in 2017 followed up with 4,214 participants, 92.1% of the original panel. The third survey in 2018 followed up with 4,104 participants (89.7% of the original panel). Of the 4,577 participants initially surveyed, individuals with incomplete data were excluded: 544 individuals who lacked information on self-efficacy, self-esteem, and health risk and behaviors.

### Independent variables

There were two independent variables studied: self-efficacy and self-esteem. These are discussed in the following sections.

#### Self-efficacy

Self-efficacy was assessed through ten statements. Participants were asked to rate 10 statements from 1–4, wherein 1 indicated that they strongly disagreed with the statement, 2 that they disagreed, 3 that they agreed, and 4 that they strongly agreed. Scores per item were summated, with the total possible score ranging from 10–40 points. Afterward, scores were categorized into four groups: 19 or less, 20 to 24, 25 to 29, and 30 or more. The items were as follows: “1. I can accomplish difficult tasks if I try hard enough;” “2. It is easy for me to focus on a goal and achieve it;” “3. I believe in my abilities, so I don’t panic when I face difficulties;” “4. I can solve most problems if I make the necessary effort; “5. Even if there are difficulties, I will be able to find a solution;” “6. I believe that even unexpected things will be done efficiently;” “7. Thanks to my talent, I know how to deal with unexpected situations;” “8. When I run into a problem, I usually find some solutions;” “9. Even if someone disagrees with my opinion, I will be able to find a way to do things the way I want;” and “10. No matter what happens to me, I will be able to solve it.”

#### Self-esteem

Self-esteem was assessed through ten statements. Participants were asked to rate 10 statements from 1–4, wherein 1 indicated that they strongly disagreed with the statement, 2 meant they disagreed, 3 meant they agreed, and 4 meant they strongly disagreed. Scores per item were summated, with the total possible score ranging from 10–40 points. Afterward, scores were categorized into four groups: 19 or less, 20 to 24, 25 to 29, and 30 or more. The items were as follows: “1. I think I’m a worthy person like others;” “2. I think I have good character;” “3. I think I’m mostly a failed person;” “4. I can do as well as others;” “5. I have little to boast;” “6. I have a positive attitude towards myself;” “7. I am generally satisfied with myself;” “8. I wish I could respect myself more;” “9. I sometimes feel like I’m a useless person;” “10. I sometimes think I am a bad person.”

### Dependent variables

#### Self-report of depression

Self-report of depression were assessed with the question: “Have you experienced depression in the past year?” Participants answered with either “yes” or “no.”

#### Perceived stress

Perceived stress was assessed with the question: “How much stress do you feel in everyday life?” Items were dichotomized for analysis using a generalized estimating equation (GEE) model with binary outcomes. The responses “Do not feel stress at all,” “I don’t feel stress,” or “An average amount of stress” were interpreted as “No stress,” while the responses “I feel it,” and “I feel it very much,” were interpreted as “Stress.”

### Control variables

#### Socioeconomic and demographic factors

Gender was categorized as male or female. There were five age groups: 15–29, 30–39, 40–49, 50–59, and ≥60 years old. Residential regions were categorized into metropolitan (Seoul), urban (Daejeon, Daegu, Busan, Incheon, Kwangju, or Ulsan), and rural (otherwise) areas. Marital status was divided into three groups: married, separated or divorced, and single. Finally, economic activity status was divided into five groups: wage workers, self-employed, unpaid family volunteers, unemployed, non-economic activity.

#### Health status and behavioral factors

Smoking status and alcohol consumption were divided into three groups: current users, former users, and those who never used. Health status was self-reported by answering the question “How do you usually perceive your health?” The responses “insufficient” and “very insufficient” were interpreted as “Bad,” while the responses “normal,” “sufficient,” or “very sufficient” were interpreted as “Good.” In Korea, disability is categorized into six levels; the first to third disability ratings generally indicate severe disability, while the rest indicate moderate levels of disability.

#### Analytical approach and statistics

The chi-square test and GEE model were used to investigate the association between self-efficacy and self-esteem and mental health. The benefit of having panel data analysis such as GEE is that it can control for time-invariant, unobservable differences between individuals. GEE models allow for substantial flexibility in specifying the correlation structure within multiple observations per individual. The GEE model also evaluated the unbalanced data relative to correlated outcomes over time. To determine whether the probability of psychological distress changed over time, we included time (year) in the model as a categorical covariate; the regression coefficient was used to estimate the annual change in both the probability of psychological distress and independent variables. For all analyses, statistical significance was set to *p* <0.05, two-tailed. All analyses were conducted using the SAS statistical software package, version 9.4 (SAS Institute Inc., Cary, NC, USA).

## Results

### Prevalence of depression

Table 1 displays the descriptive statistics of all variables during the baseline analysis in 2016. Of the 4,033 research participants included in 2016, those who felt stress or depression were 41.7% (1,681 participants) and 18.0% (725 participants), respectively. The proportion of participants with self-esteem scores ≥30 was 22.0% (886 participants); of those with self-esteem scores ≥30, the proportion of participants who felt stress or depression was 438 (49.4%) and 74 (8.4%), respectively. Finally, the proportion of those with self-efficacy scores ≥30 was 38.7% (1,561 participants); among these participants, 681 (43.6%) and 187 (12.0%), felt stress and depression respectively. (Table 1)

**Table 1:** General characteristics of subjects included for analysis.

	Total		Stress		P-value	Self-report of depression		P-value
	N	%	Yes	%		Yes	%	
**Self-esteem**					< .0001			< .0001
**≤19**	107	2.7	29	27.1		63	58.9	
**20–24**	836	20.7	271	32.4		247	29.6	
**25–29**	2,204	54.7	943	42.8		341	15.5	
**≥30**	886	22.0	438	49.4		74	8.4	
**Self-efficacy**					0.045			< .0001
**≤19**	566	14.0	236	41.7		181	32.0	
**20–24**	814	20.2	306	37.6		192	23.6	
**25–29**	1,092	27.1	458	41.9		165	15.1	
**≥30**	1,561	38.7	681	43.6		187	12.0	
**Gender**					0.084			0.016
**Male**	2,636	65.4	1,563	59.3		446	16.9	
**Female**	1,397	34.6	789	56.5		279	20.0	
**Age**					0.001			< .0001
**15–29**	700	17.4	328	46.9		92	13.1	
**30–39**	978	24.3	427	43.7		151	15.4	
**40–49**	1,160	28.8	431	37.2		214	18.5	
**50–59**	783	19.4	325	41.5		185	23.6	
**≥60**	412	10.2	170	41.3		83	20.2	
**Residential region**					0.154			0.406
**Metropolitan**	783	19.4	324	41.4		147	18.8	
**Urban**	1,183	29.3	520	44.0		198	16.7	
**Rural**	2,067	51.3	837	40.5		380	18.4	
**Marital status**					0.014			< .0001
**Married**	1,809	44.9	724	40.0		223	12.3	
**Separated, divorced**	1,635	40.5	726	44.4		312	19.1	
**Single**	589	14.6	231	39.2		190	32.3	
**Economic activity status**								< .0001
**Wage workers**	1,560	38.7	683	43.8		168	10.8	
**Self-employed**	389	9.7	149	38.3		39	10.0	
**Unpaid family volunteers**	67	1.7	24	35.8		8	11.9	
**Unemployed**	145	3.6	64	44.1		35	24.1	
**Non-economic activity**	1,872	46.4	761	40.7		475	25.4	
**Smoking status**					< .0001			0.005
**Current smoker**	912	22.6	325	35.6		192	21.1	
**Former smoker**	773	19.2	307	39.7		148	19.2	
**Nothing**	2,348	58.2	1,049	44.7		385	16.4	
**Alcohol consumption**					< .0001			< .0001
**Current drinker**	1,686	41.8	674	40.0		267	15.8	
**Former drinker**	773	19.2	287	37.1		184	23.8	
**Nothing**	1,574	39.0	720	45.7		274	17.4	
**Self-rated health**					< .0001			< .0001
**Bad**	1,880	46.6	633	33.7		527	28.0	
**Good**	2,153	53.4	1,048	48.7		198	9.2	
**Self-report of depression**					< .0001			
**Yes**	725	18.0	141	19.5				
**No**	3,308	82.0	1,540	46.6				
**Stress**								< .0001
**Yes**	1,681	41.7				141	8.4	
**No**	2,352	58.3				584	24.8	
**Disability level**					0.202			< .0001
**Severe (1,2,3)**	1,556	38.6	668	42.9		339	21.8	
**Moderate(others)**	2,477	61.4	1,013	40.9		386	15.6	
**Total**	4,033	100.0	1,681	41.7		725	18.0	

### Association between self-esteem and self-efficacy and self-report of depression

Table 2 shows the relationship between self-esteem and self-efficacy and self-report of depression; the relationships have been adjusted for socioeconomic status, health risk, and health status factors. After adjusting for all of these confounders (Model 3), the odds of having self-report of depression in those with self-esteem scores ≤19 was 5.825 times higher (95% Confidence Interval [CI] = 4.235–8.011; *p* < .0001) than those with self-esteem scores ≥30. The odds of having self-report of depression in those with self-esteem scores from 20 to 24 were 2.676 times higher (95% CI = 2.173–3.296; *p* < .0001) than those with self-esteem scores ≥30. Meanwhile, the odds of having self-report of depression in those with self-efficacy scores ≤19 was 1.494 times higher (95% CI = 1.233–1.810; *p* < .0001) than those with self-efficacy scores ≥30. Finally, the odds of having self-report of depression in those with self-efficacy scores from 20 to 24 was 1.371 times higher (95% CI = 1.161–1.619; *p* = .0001) than those with self-efficacy scores ≥30 (Table 2).

**Table 2:** Association between self-evaluation and self-report of depression.

	Model 1				Model 2				Model 3			
	*OR*	95% CI		P-value	*OR*	95% CI		P-value	*OR*	95% CI		P-value
**Self-esteem**												
**≤19**	7.241	5.347	9.806	< .0001					5.825	4.235	8.011	< .0001
**20–24**	3.034	2.484	3.705	< .0001					2.676	2.173	3.296	< .0001
**25–29**	1.890	1.579	2.262	< .0001					1.782	1.483	2.141	< .0001
**≥30**	1.000								1.000			
**Self-efficacy**												
**≤19**					2.251	1.883	2.692	< .0001	1.494	1.233	1.810	< .0001
**20–24**					1.712	1.458	2.010	< .0001	1.371	1.161	1.619	0.000
**25–29**					1.161	0.998	1.350	0.054	1.040	0.891	1.213	0.619
**≥30**					1.000				1.000			
**Gender**												
**Male**	1.000				1.000				1.000			
**Female**	1.422	1.229	1.646	< .0001	1.421	1.230	1.643	< .0001	1.439	1.243	1.666	< .0001
**Age**												
**15–29**	0.798	0.617	1.032	0.086	0.778	0.602	1.004	0.054	0.819	0.633	1.060	0.129
**30–39**	1.178	0.953	1.455	0.130	1.212	0.983	1.496	0.072	1.203	0.973	1.487	0.088
**40–49**	1.087	0.900	1.313	0.386	1.155	0.958	1.394	0.132	1.108	0.917	1.340	0.287
**50–59**	1.183	0.982	1.424	0.077	1.230	1.022	1.479	0.028	1.197	0.993	1.442	0.059
**≥60**	1.000				1.000				1.000			
**Residential region**												
**Metropolitan**	1.000				1.000				1.000			
**Urban**	0.809	0.686	0.953	0.011	0.767	0.652	0.903	0.001	0.789	0.669	0.931	0.005
**Rural**	1.115	0.961	1.294	0.151	1.067	0.921	1.236	0.385	1.093	0.941	1.268	0.245
**Marital status**												
**Married**	1.000				1.000				1.000			
**Separated, divorced**	1.501	1.286	1.750	< .0001	1.563	1.342	1.822	< .0001	1.443	1.236	1.685	< .0001
**Single**	1.768	1.517	2.061	< .0001	1.845	1.585	2.148	< .0001	1.734	1.487	2.022	< .0001
**Economic activity status**												
**Wage workers**	0.571	0.493	0.661	< .0001	0.562	0.485	0.652	< .0001	0.615	0.529	0.714	< .0001
**Self-employed**	0.596	0.473	0.751	< .0001	0.569	0.451	0.719	< .0001	0.648	0.513	0.820	0.000
**Unpaid family volunteers**	0.414	0.242	0.707	0.001	0.404	0.237	0.689	0.001	0.426	0.249	0.728	0.002
**Unemployed**	1.123	0.833	1.513	0.446	1.166	0.866	1.568	0.312	1.206	0.894	1.628	0.220
**Non-economic activity**	1.000				1.000				1.000			
**Smoking status**												
**Current smoker**	0.960	0.820	1.123	0.608	1.636	1.371	1.952	< .0001	1.566	1.310	1.871	< .0001
**Former smoker**	1.090	0.930	1.279	0.287	1.340	1.117	1.608	0.002	1.296	1.078	1.557	0.006
**Nothing**	1.000				1.000				1.000			
**Alcohol consumption**												
**Current drinker**	1.545	1.294	1.846	< .0001	1.007	0.860	1.179	0.927	0.992	0.846	1.163	0.921
**Former drinker**	1.278	1.064	1.535	0.009	1.140	0.972	1.336	0.107	1.128	0.960	1.324	0.143
**Nothing**	1.000				1.000				1.000			
**Self-rated health**												
**Bad**	2.397	2.108	2.725	< .0001	2.426	2.133	2.760	< .0001	2.294	2.014	2.613	< .0001
**Good**	1.000				1.000				1.000			
**Stress**												
**No**	1.000				1.000				1.000			
**Yes**	3.091	2.727	3.504	< .0001	3.250	2.870	3.681	< .0001	3.093	2.728	3.506	< .0001
**Disability level**												
**Severe (1,2,3)**	1.186	1.054	1.336	0.005	1.151	1.021	1.298	0.022	1.125	0.996	1.270	0.058
**Moderate(others)**	1.000				1.000				1.000			
**Year**												
**2016**	1.228	1.075	1.402	0.003	1.252	1.097	1.428	0.001	1.219	1.067	1.393	0.004
**2017**	1.037	0.904	1.189	0.603	1.050	0.917	1.203	0.478	1.037	0.904	1.190	0.605
**2018**	1.000				1.000				1.000			

### Association between self-esteem and self-efficacy and perceived stress

Table 3 shows the relationship between self-esteem and self-efficacy and perceived stress adjusted for socioeconomic status, health risk, and health status factors. After adjusting for all of these confounders (Model 3), we determined that those with self-esteem scores ≤19 were 2.036 times more likely to experience perceived stress (95% CI: 1.510–2.747 p-value: < .0001) than those with self-esteem scores ≥30. The odds of perceived stress among those with self-esteem scores from 20 to 24 were 1.451 times higher (95% CI: 1.269–1.659 p-value: < .0001) than those with self-esteem scores ≥30 (Table 3).

**Table 3:** Association between self-evaluation and perceived stress.

	Model 1				Model 2				Model 3			
	*OR*	95% CI		P-value	*OR*	95% CI		P-value	*OR*	95% CI		P-value
**Self-esteem**												
**≤19**	1.855	1.391	2.473	< .0001					2.036	1.510	2.747	< .0001
**20–24**	1.442	1.268	1.640	< .0001					1.451	1.269	1.659	< .0001
**25–29**	1.112	1.013	1.220	0.026					1.097	0.997	1.207	0.059
**≥30**	1.000								1.000			
**Self-efficacy**												
**≤19**					1.016	0.882	1.170	0.830	0.865	0.745	1.005	0.058
**20–24**					1.176	1.044	1.324	0.008	1.090	0.964	1.232	0.168
**25–29**					1.109	1.009	1.219	0.033	1.074	0.975	1.183	0.150
**≥30**					1.000				1.000			
**Gender**												
**Male**	1.000				1.000				1.000			
**Female**	1.041	0.941	1.151	0.437	1.024	0.927	1.133	0.636	1.036	0.936	1.145	0.498
**Age**												
**15–29**	1.697	1.422	2.026	< .0001	1.681	1.408	2.006	< .0001	1.706	1.429	2.038	< .0001
**30–39**	1.357	1.172	1.572	< .0001	1.371	1.184	1.588	< .0001	1.364	1.178	1.580	< .0001
**40–49**	1.423	1.244	1.627	< .0001	1.447	1.265	1.654	< .0001	1.429	1.250	1.635	< .0001
**50–59**	1.107	0.967	1.267	0.142	1.119	0.977	1.280	0.104	1.107	0.967	1.267	0.142
**≥60**	1.000				1.000				1.000			
**Residential region**												
**Metropolitan**	1.000				1.000				1.000			
**Urban**	0.967	0.864	1.082	0.555	0.963	0.860	1.077	0.508	0.967	0.864	1.082	0.560
**Rural**	0.987	0.891	1.093	0.800	0.982	0.887	1.089	0.735	0.990	0.893	1.097	0.841
**Marital status**												
**Married**	1.000				1.000				1.000			
**Separated, divorced**	0.748	0.672	0.832	< .0001	0.769	0.691	0.856	< .0001	0.753	0.676	0.838	< .0001
**Single**	0.832	0.738	0.937	0.003	0.846	0.751	0.953	0.006	0.830	0.737	0.936	0.002
**Economic activity status**												
**Wage workers**	1.214	1.101	1.339	0.000	1.166	1.056	1.287	0.002	1.206	1.092	1.333	0.000
**Self-employed**	1.546	1.334	1.791	< .0001	1.479	1.275	1.717	< .0001	1.541	1.328	1.790	< .0001
**Unpaid family volunteers**	1.298	0.972	1.733	0.077	1.260	0.944	1.682	0.117	1.285	0.963	1.716	0.089
**Unemployed**	1.353	1.066	1.718	0.013	1.304	1.026	1.656	0.030	1.328	1.045	1.688	0.021
**Non-economic activity**	1.000				1.000				1.000			
**Smoking status**												
**Current smoker**	1.309	1.160	1.478	< .0001	1.321	1.171	1.491	< .0001	1.308	1.158	1.476	< .0001
**Former smoker**	1.047	0.928	1.182	0.452	1.054	0.934	1.189	0.396	1.046	0.927	1.180	0.464
**Nothing**	1.000				1.000				1.000			
**Alcohol consumption**												
**Current drinker**	1.183	1.064	1.315	0.002	1.180	1.061	1.311	0.002	1.175	1.057	1.307	0.003
**Former drinker**	1.125	1.002	1.262	0.046	1.116	0.994	1.253	0.063	1.113	0.991	1.250	0.070
**Nothing**	1.000				1.000				1.000			
**Self-rated health**												
**Bad**	1.682	1.540	1.836	< .0001	1.721	1.575	1.880	< .0001	1.689	1.545	1.845	< .0001
**Good**	1.000				1.000				1.000			
**Self-report of depression**												
**Yes**	3.078	2.718	3.486	< .0001	3.258	2.879	3.688	< .0001	3.096	2.733	3.507	< .0001
**No**	1.000				1.000				1.000			
**Disability level**												
**Severe (1,2,3)**	0.849	0.779	0.925	0.000	0.867	0.794	0.947	0.002	0.861	0.789	0.940	0.001
**Moderate(others)**	1.000				1.000				1.000			
**Year**												
**2016**	1.111	1.012	1.219	0.027	1.117	1.017	1.225	0.020	1.107	1.008	1.215	0.033
**2017**	1.079	0.983	1.184	0.108	1.079	0.983	1.183	0.109	1.077	0.981	1.181	0.118
**2018**	1.000				1.000				1.000			

## Discussion

In this study, we aimed to investigate the association between self-evaluations, such as self-efficacy and self-esteem, and mental health in a longitudinal model using a nationally representative sample of the population of people with disabilities in South Korea. The present study suggests that people with disabilities who have low self-evaluation in both self-esteem and self-efficacy have a higher risk of exhibiting self-report of depression and perceived stress compared to those with higher self-evaluations. To the best of our knowledge, this is the first epidemiological study wherein self-esteem and self-efficacy were appraised simultaneously using a longitudinal database of a large representative sample of people with disabilities; additionally, we believe this is the first epidemiological study correlating this information with mental health.

Our findings that indicate an inverse correlation between self-evaluation and mental health are supported by existing literature. Declining self-evaluation was associated with a risk for poor mental health. These were consistent with published studies that have found that individuals with low self-evaluation also report negative experiences—such as increased fatigue and pain [19]—and that have previously demonstrated associations between self-evaluation and mental health [20, 21]. These effects may be explained by daily life dissatisfaction due to physical or/and mental disabilities particularly among people with disabilities who have low self-evaluations since these individuals are prone to set low goals for themselves. Disappointment and helplessness during daily life could foster a decline in perceived self-evaluation and an increased risk of poor mental health. Consequently, poor mental health may negatively affect perceived self-evaluation and further impede performance [22].

Existing literature also supports our assertion that self-evaluations, such as self-efficacy and self-esteem, influence the thought patterns and emotional reactions of an individual. As previously noted [23], significant correlations were found among helplessness, cognitive distortions, and self-efficacy. That is, cognitive distortions may cause feelings of helplessness and low self-efficacy, and, in turn, have indirect effects on depression. Additionally, Bandura and colleagues also found that human depression may be generated through cognition, dejecting ruminative thoughts. Feeling that one has little ability to exercise control over these ruminative thoughts also contributes to the development of depression [14].

Several observations are noteworthy. Learned helplessness and self-efficacy theories arose from attempts to explain self-regulatory behavioral phenomena. Scheier and Carver [24] posited that individuals who see desired outcomes as attainable continue to exert efforts to attain those outcomes, even when doing so is difficult. Lenker et al. [25] further posited that individuals who feel in control of their illness may be self-efficacious in carrying out self-management techniques for themselves.

There are several limitations related to the present study. First,, data was gathered from self-reports, which may have been imperfect indicators of actual behavior and can potentially be affected by false consciousness or by the adaptation of resources. Second, information on health status and risk behavior factors was insufficient. There might also have been unobserved confounders. Consequently, the lack of such information might have resulted in an underestimation of the results in the present study. Nevertheless, despite the underestimation, we found a significant association between self-evaluation and mental health in people with disabilities; further research is needed to understand the underlying mechanisms contributing to such poor self-evaluation and mental health among people with disabilities.

Despite these limitations, this study has various strengths, particularly with its use of a population-based representative sample and the 3-year follow-up database. This study made use of longitudinal data from a large number of participants who were representative of the overall South Korean population of people with disabilities. Given that the sample size was large, the results may be generalized to the rest of the nation. Based on these results, health care givers can reduce depressive symptoms and stress by managing the self-efficacy and self-esteem of the people with disability.

## Conclusion

In conclusion, the findings of this study indicate that there exists an inverse correlation between self-evaluations, such as self-efficacy and self-esteem, and mental health in people with disabilities. It can be concluded from that high self-evaluation among these people may with disabilities serve as a protective factor against poor mental health; conversely, low self-evaluation can lead to poor mental health. The results of this study can be used as a basis for developing intervention strategies and training and intervention programs for people with disabilities. As such, efforts should be made to enable these people with disabilities to learn how to face the challenges of life with courage.
