# Correction: Association between self-esteem and efficacy and mental health in people with disabilities

**DOI:** 10.1371/journal.pone.0303430

**Published:** 2024-05-03

**Authors:** The PLOS ONE Editors

There are errors in the Author Contributions of the first author, Jong Youn Moon. The correct contributions are: Investigation, Validation, Writing–review & editing.

In addition, the Funding statement is incorrect. The correct statement is: The authors received no specific funding for this work.
